# Supplementary figures and images for: Expression analysis of genes related to cold tolerance in Dendroctonus valens
Source: PeerJ. 2021 Mar 9;9:e10864. doi: 10.7717/peerj.10864 (PMC7953874; doi:10.7717/peerj.10864)

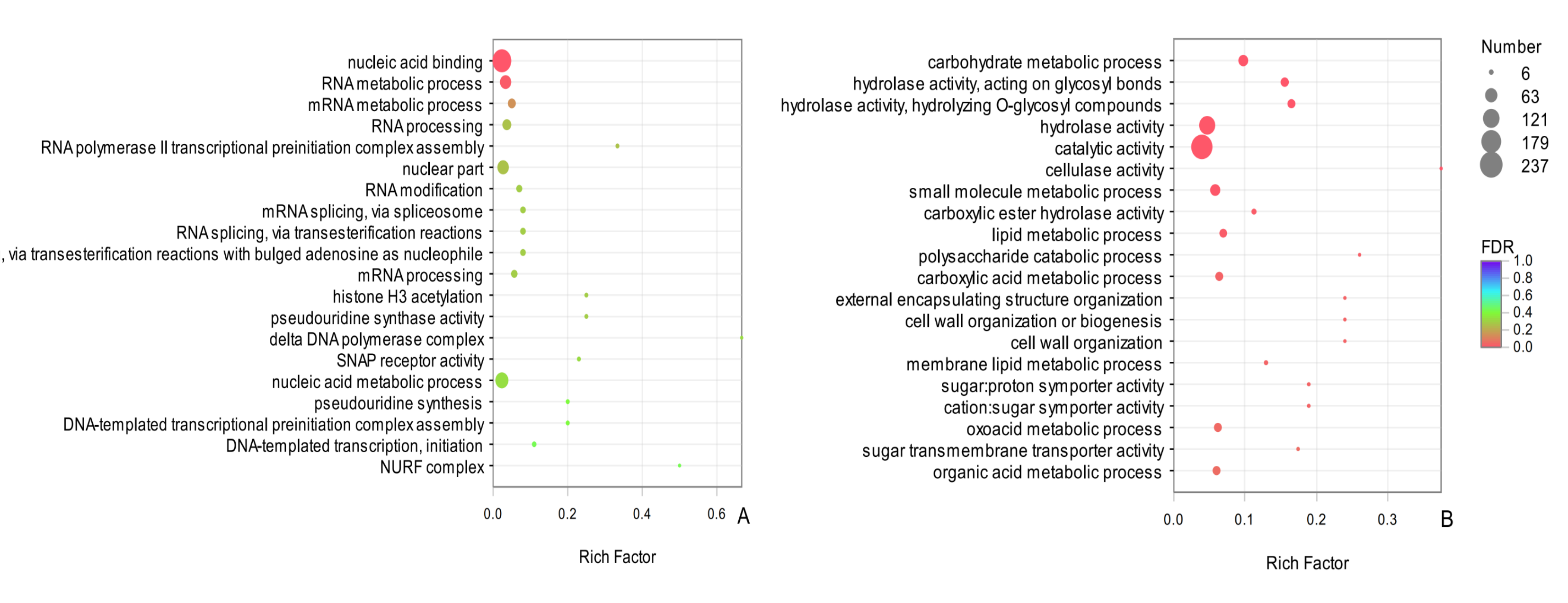

Supplement: Figure S1 — The vertical axis represents the pathway name, and the horizontal axis represents the Rich factor. The larger the Rich factor, the greater the degree of enrichment, the size of the dots indicates how many genes are in this pathway, and the colors of the dots correspond to different Qvalue ranges. Red indicates significant enrichment. [file peerj-09-10864-s001.png]

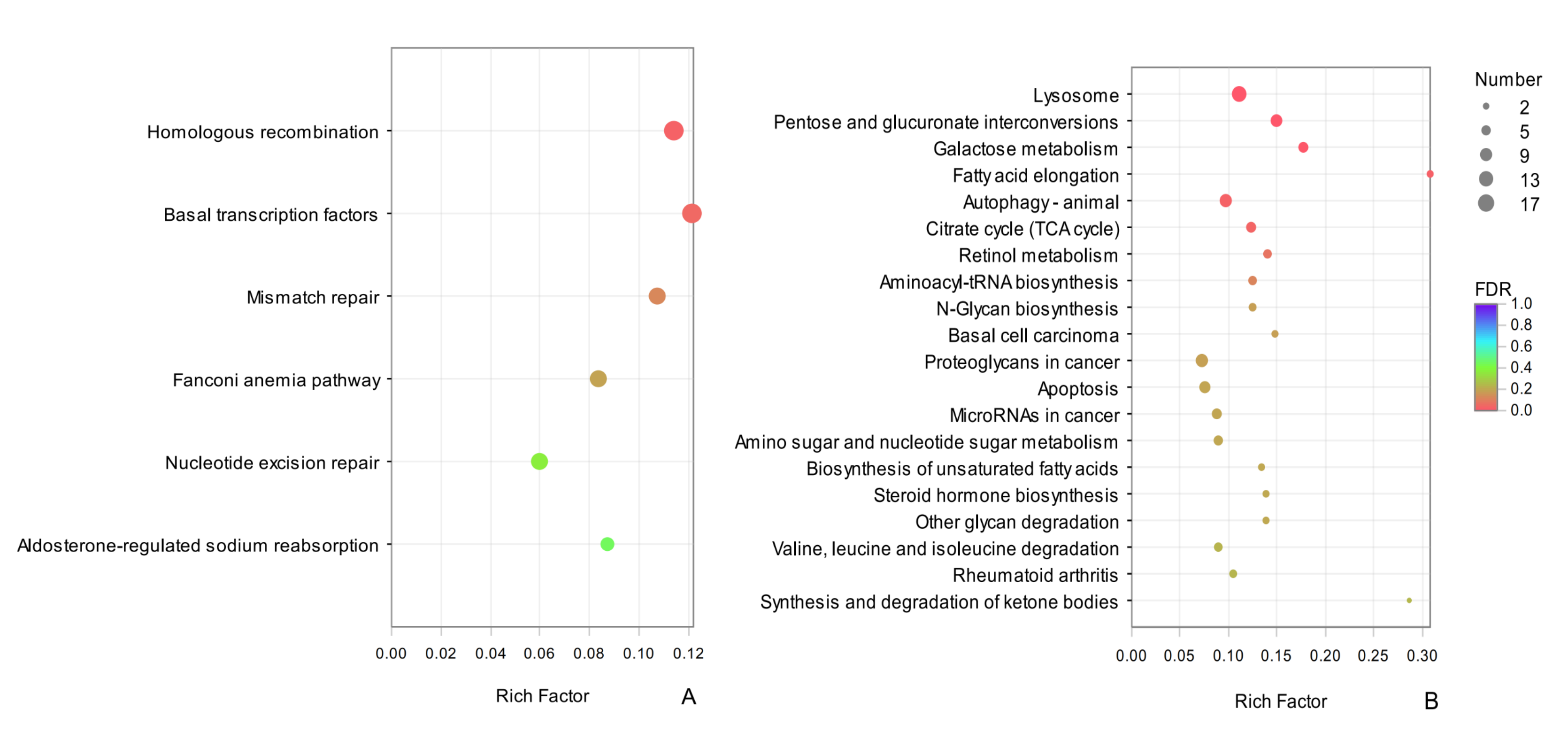

Supplement: Figure S2 — The vertical axis represents the pathway name, and the horizontal axis represents the rich factor. The larger the rich factor, the greater the degree of enrichment, the size of the dots indicates how many genes are in this pathway, and the colors of the dots correspond to different Qvalue ranges. Red indicates significant enrichment. [file peerj-09-10864-s002.png]
