# Supplementary material for: Expression analysis of genes related to cold tolerance in Dendroctonus valens
Source: PeerJ. 2021 Mar 9;9:e10864. doi: 10.7717/peerj.10864 (PMC7953874; doi:10.7717/peerj.10864)
Supplement: Table S1 [file peerj-09-10864-s003.docx]

Table S1: The quantitative PCR primers of candidate genes in *D. valens*

| **Gene ID** | **Primer sequences（5’-3’）** | **Product length（bp）** | **Primer-pair efficiency（%）** | **R^2^** |
| --- | --- | --- | --- | --- |
| TRINITY_DN22243_c0_g1 | F:TCCATGGATGTTTGAGGCCC R:TCTGACGACTGTTGCCAGAC | 187 | 109.4 | 0.997 |
| TRINITY_DN18757_c0_g2 | F:TCAACGTCGGTCTGTCTGTG  R:ACGTACTGGCCCTGTTTGAG | 208 | 105.5 | 0.997 |
| TRINITY_DN26187_c0_g1 | F:GGGCGAGGACAAATCAGGAA  R:AGCCATTTCAGAGGGAAGGC | 160 | 96.7 | 0.996 |
| TRINITY_DN21780_c1_g1 | F:TGGCCGAACAGACCAAAGTT  R:ATGAGCAAAGCCACCGTGTA | 235 | 94.2 | 0.998 |
| TRINITY_DN22610_c0_g2 | F:TTATGACGACTGTCCACGCC  R:TGCCAGTCAATTTGCCGTTG | 171 | 99.5 | 0.998 |
| TRINITY_DN27207_c0_g1 | F:GCAATGAATAAGCTGGGCGG  R:AGATCATCAGCGTGTGCCAA | 152 | 104.4 | 0.993 |
| TRINITY_DN27539_c3_g1 | F:ATTGCATCCTGGGGTTGGAG  R:CGTAGTAGCTGGTGCCGTAG | 186 | 93.4 | 0.993 |
| TRINITY_DN18927_c0_g1 | F:AAACTTTGGATGCGATGGCG  R:ACAGCGCTTCTTCATCACCA | 182 | 96.3 | 0.999 |
| TRINITY_DN22377_c1_g1 | F:TCCACGTCGATCTGCAAACA  R:GCCATCGCAATGTCTTCGTC | 208 | 99.2 | 0.994 |
| TRINITY_DN26090_c0_g1 | F:TGCAACTCTGGCGGAAATCT  R:CGATGTTGCCGTTGACGATC | 158 | 94.5 | 0.991 |
| TRINITY_DN19691_c0_g1 | F:ACGCCACTGACCTGGAAAAT  R:TCCCAGATGTTGATTGCGCT | 214 | 105.9 | 0.995 |
| TRINITY_DN25425_c0_g4 | F:AATGTGTGTAGCTGACGCCA  R:GCGTCTCATCAAACCTCCCA | 163 | 108.5 | 0.994 |
| TRINITY_DN27537_c0_g1 | F:TTGAAGACTCACCGAACGGG  R:TGCTGGCAACCCAAATAGCT | 158 | 99.8 | 0.993 |
| TRINITY_DN20229_c0_g1 | F:TGCAGTGGAGATTGGAGCAG  R:TGTTGACGCCATTAGCCACT | 237 | 96.9 | 0.999 |
| TRINITY_DN22439_c0_g5 | F:CTGCGGAAATCGAATCTGGC  R:GTCTTGGGGAGTTGGGACAG | 185 | 102.3 | 0.998 |
| *TUB* | F:CTTACCACCCCCACATACGG  R:ATTGCTGACTGCCTCTGGAC | 179 | 97.8 | 1.000 |
| *PRS18* | F:CATCGCTCTGTCCTCGGTAC  R:TCGGTGTGCTTGACATCCAA | 216 | 102.3 | 0.999 |
