# Supplementary material for: Expression analysis of genes related to cold tolerance in Dendroctonus valens
Source: PeerJ. 2021 Mar 9;9:e10864. doi: 10.7717/peerj.10864 (PMC7953874; doi:10.7717/peerj.10864)
Supplement: Table S2 [file peerj-09-10864-s004.docx]

**Table S2：Assembly statistics of *D. valens* transcriptome**

| **Type** | **Resource** |
| --- | --- |
| Total transcripts num | 90404 |
| Total unigenes num | 50677 |
| Total sequence base | 91263286 |
| Largest | 30967 |
| Smallest | 201 |
| Average length | 911.08 |
| N50 | 1803 |
| GC percent | 40.17 |
| BUSCO score | C:89.2%[S:84.9%,D:4.3%], F:6.9%, M:3.9%, n:978 |
